# Effects of shakuyakukanzoto and its absorbed components on twitch contractions induced by physiological Ca^2+^ release in rat skeletal muscle

**DOI:** 10.1007/s11418-015-0890-z

**Published:** 2015-03-18

**Authors:** Noriko Kaifuchi, Yuji Omiya, Hirotaka Kushida, Miwako Fukutake, Hiroaki Nishimura, Yoshio Kase

**Affiliations:** 1 Tsumura Research Laboratories, Kampo Scientific Strategies Division, Tsumura & Co., 3586 Yoshiwara, Ami-machi, Inashiki-gun, Ibaraki, 300-1192 Japan; 2 Production Division, Kampo Formulations Development Center, Tsumura & Co., 3586 Yoshiwara, Ami-machi, Inashiki-gun, Ibaraki, 300-1192 Japan

**Keywords:** Shakuyakukanzoto, *Kampo*, *Glycyrrhizae* radix, *Paeoniae* radix, Skeletal muscle

## Abstract

Shakuyakukanzoto (SKT) is a *kampo* medicine composed of equal proportions of *Glycyrrhizae* radix (*G*. radix) and *Paeoniae* radix (*P*. radix). A double-blind study reported that SKT significantly ameliorated painful muscle cramp in cirrhosis patients without the typical severe side effects of muscle weakness and central nervous system (CNS) depression. Previous basic studies reported that SKT and its active components induced relaxation by a direct action on skeletal muscle and that SKT did not depress CNS functions; however, why SKT has a lower incidence of muscle weakness remains unknown. In the present study, we investigated which components are absorbed into the blood of rats after a single oral administration of SKT to identify the active components of SKT. We also investigated the effects of SKT and its components on the twitch contraction induced by physiological Ca^2+^ release. Our study demonstrated that SKT and five *G*. radix isolates, which are responsible for the antispasmodic effect of SKT, did not inhibit the twitch contraction in contrast to dantrolene sodium, a direct-acting peripheral muscle relaxant, indicating that the mechanisms of muscle contraction of SKT and dantrolene in skeletal muscle differ. These findings suggest that SKT does not reduce the contractile force in skeletal muscle under physiological conditions, i.e., SKT may have a low risk of causing muscle weakness in clinical use. Considering that most muscle relaxants and anticonvulsants cause various harmful side effects such as weakness and CNS depression, SKT appears to have a benign safety profile.

## Introduction

Most relaxants or anticonvulsants commonly work systemically against the symptoms of muscle pain and spasms; their therapeutic effects, however, are not always sufficient. Furthermore, these drugs produce adverse effects such as muscle weakness and central nervous system (CNS) depression, including dizziness and drowsiness [1, 2], which impair the quality of life of patients.

Shakuyakukanzoto (SKT) is a *kampo* medicine composed of equal proportions of *Glycyrrhizae* radix (*G*. radix) and *Paeoniae* radix (*P*. radix). Clinical studies to date have reported that SKT significantly ameliorates muscle symptoms in patients with a broad array of underlying diseases. SKT has shown immediate efficacy on painful muscle cramps induced by liver cirrhosis [3], diabetic neuropathy [4], and hemodialysis [5, 6]. In addition, SKT has been found to ameliorate the myalgia and arthralgia induced by combination paclitaxel and carboplatin chemotherapy [7–9]. It is well known that a habitual daily intake and excessive daily ingestion of *G*. radix induce pseudoaldosteronism, which is characterized by hypokalemia and rhabdomyolysis [10]. However, a double-blind study in which the patients with liver cirrhosis received a daily dose of 7.5 g SKT in three divided doses for 2 weeks reported that there was no significant difference in the frequencies of adverse reactions between SKT and placebo groups, and that SKT did not induce severe adverse effects such as muscle weakness and CNS depression [3]. Other clinical studies have further reported that no adverse events were observed during treatment with SKT [5–7, 9].

A recent study found that SKT (intraduodenal administration, i.d.) and six *G*. radix isolates (intravenous administration, i.v.) [glycyrrhetic acid (GA), liquiritigenin (LQG), liquiritin apioside (LQA), isoliquiritigenin (ILQG), isoliquiritin apioside (ILQA), and glycycoumarin (GCM)] act directly on skeletal muscles to inhibit tetanic contractions with a mechanism of action outside the CNS in an experimental cramp model [11]. Moreover, a basic pharmacological study revealed that SKT (2 g/kg) does not affect general behavior nor significantly change the functions of CNS, respiratory, cardiovascular, gastrointestinal, or renal systems in normal animals [12]. However, why SKT treatment has a lower incidence of muscle weakness, which is known as a typical adverse effect of muscle relaxants, remains to be revealed.

Moreover, since the pharmacokinetic profiles of active components in SKT after oral administration have not been adequately investigated, it remains unclear whether those constituents are actually absorbed into the systemic circulation to exert the effects on skeletal muscle. In the present study, therefore, we investigated which components are absorbed into the systemic circulation after a single oral administration of SKT to rats first. Secondly, we investigated the effects of SKT and its absorbed components on twitch contractions induced by electrical stimulation of intact skeletal muscle cell. Dantrolene sodium, a well-known direct-acting peripheral muscle relaxant, was used as a control drug.

## Materials and methods

### Animals

Male Sprague–Dawley and Wistar rats were purchased from Japan SLC (Shizuoka). Sprague–Dawley rats were used to identify the absorbed components of SKT in accordance with previous studies [14–16]. Because a previous study reported that isometric training induced muscle hypertrophy of the gastrocnemius in Sprague–Dawley rats [17], the effects of SKT and its absorbed components on electrically induced muscle contraction were studied using Wistar rats. All animal experiments were conducted in accordance with the guidelines for animal use and care established by the Laboratory Animal Committee, Tsumura & Co. (Tokyo).

### SKT and its components

The spray-dried powder extracts of SKT (nos. 280068010, 322098500) were supplied by the Ibaraki Plant, Tsumura & Co. SKT was composed of equal proportions of *G*. radix (root and stolon of *Glycyrrhiza uralensis* Fischer, Leguminosae) and *P*. radix (root of *Paeonia lactiflora* Pallas, Paeoniaceae), which was manufactured in compliance with the Japanese Pharmacopoeia (Sixteenth Edition, JP16) under good manufacturing practice (GMP). Therefore, the quality and authenticity of SKT extract powder were assured. SKT was prepared by decocting *G*. radix and *P*. radix in boiling water for 60 min. The extraction yield of SKT was approximately 21 %. The voucher specimens have been deposited at the Ibaraki Plant, Tsumura & Co. The extraction procedure and chemical profiles of SKT have been reported in a previous study [11]. Briefly, 1.0 g of SKT was extracted with 20 mL of 75 % methanol for 30 min for high-performance liquid chromatography-ultraviolet (HPLC-UV) analysis. Chemical profiles of SKT were investigated using the three-dimensional HPLC fingerprint method.

The powdered extracts of GA, liquiritin (LQ), LQG, LQA, isoliquiritin (ILQ), ILQG, ILQA, GCM, paeoniflorin (PAE), and albiflorin (ALB) with purities high enough for use in biological studies were supplied by the Kampo Formulations Development Center, Tsumura & Co.

### Drug administration and sampling for pharmacokinetics investigation

Male Sprague–Dawley rats (7 weeks old, *n* = 4–5/time point) were housed for 5 days in a controlled environment with free access to water and normal chow prior to experiments. SKT was suspended in distilled water at 10 mL/kg. After a 15–17-h fast, rats received a single oral gavage dose of 1 g/kg. Blood was collected from the ventral aorta under isoflurane anesthesia at 0 (pre), 5, 15, 30 min, 1, 2, 4, 8, 10, 12, 24, 36, and 48 h after administration. Heparinized blood samples were immediately centrifuged to separate the plasma. All plasma samples were stored at −80 °C until analysis.

### Quantitative determination of plasma concentrations

We determined the plasma concentrations of eight components of *G*. radix (GA, LQ, LQG, LQA, ILQ, LQG, ILQA, and GCM) and two components of *P*. radix (PAE and ALB) in rat plasma after a single oral administration of SKT. GA, LQ, LQG, LQA, ILQ, ILQG, ILQA, GCM, PAE, and ALB were dissolved in methanol and serially diluted with 50 % aqueous methanol to make the working solutions. Calibration standards were prepared by spiking pooled blank rat plasma (Sprague–Dawley rats, Japan SLC) with the working solutions. Niflumic acid (Sigma-Aldrich, St. Louis, MO, USA) or digoxin (Alfa Aesar, Heysham, UK) was dissolved in methanol, diluted with 50 % aqueous methanol, and used as an internal standard (IS) for each analytical method.

The plasma concentrations of all components were determined using liquid chromatography-tandem mass spectrometry (LC–MS/MS). The instrument was composed of a 1260 Infinity LC System (Agilent Technologies, Santa Clara, CA, USA) and a QTRAP 5500 system (AB SCIEX, Framingham, MA, USA). Samples were prepared by protein precipitation with acetonitrile, and 5-μL aliquots were applied to the analytical column. Mass spectrometry detections were operated using an electrospray ionization (ESI) interface in negative ion mode by multiple-reaction monitoring (MRM). The calibration curve ranges (ng/mL) were 0.05–10 for GCM, 0.1–10 for ILQ, 0.1–100 for ALB, 0.5–10 for LQ, LQG, ILQG, LQA, and ILQA, 0.5–100 for PAE, and 2.0–1000 for GA. The analytical methods are summarized in Table 1.

**Table 1:** Summary of analytical determination methods for 10 components of SKT

Method	HPLC condition	MRM transition (*m*/*z*)		Target component
		Q1	Q3	
1	YMC-Pack ODS-AQ (50 × 2.0 mm, 3 μm)^a^	469.3	425.4	GA
	Gradient of 45–80 % B, over 5 min, 0.2 mL/min	281.0	237.0	Niflumic acid (IS)
	A: 10 mM NH_4_OAc in water, B: MeCN			
2	Atlantis dC18 (100 × 2.1 mm, 3 μm)^b^	417.1	255.1	LQ, ILQ
	Gradient of 10–85 % B over 17 min, 0.2 mL/min	255.0	119.0	LQG, ILQG
	A: 10 mM NH_4_OAc in water, B: MeCN	549.1	255.0	LQA, ILQA
		366.9	308.9	GCM
		780.4	650.6	Digoxin (IS)
3	Atlantis dC18 (100 × 2.1 mm, 3 μm)^b^	479.0	121.0	PAE, ALB
	Isocratic solution of A and B (82:18), 0.2 mL/min			
	A: 0.2 % AcOH in water, B: 0.2 % AcOH in MeCN			

^a^YMC (Kyoto)

^b^Waters (MA, USA)

### In vivo experiment on skeletal muscle

Male Wistar rats (250–300 g, *n* = 4–6/group) were used for in vivo contractile experiments. Experimental procedures were conducted as reported in a previous paper [11]. Briefly, the tibial nerve, which innervates the gastrocnemius muscle, on the left leg was exposed and transected on the spinal side for complete elimination of the spinal reflex in rats under urethane anesthesia (1.08 g/kg, i.p., Sigma-Aldrich). The isolated tibial nerve was connected to an electric stimulator (Nihon Kohden, Tokyo) via an isolator (Physio-Tech, Tokyo). To evoke twitch contractions, a 0.3-ms square-wave electric pulse (3 V) was applied to the tibial nerve. The contractile responses of the gastrocnemius muscle were detected by a force transducer (Nippon Avionics, Tokyo) connected to the Achilles tendon at 5, 10, 15, 20, 30, 40, 50, and 60 min after the administration of test solutions. The body temperature of rats was maintained at 37–37.5 °C during the experiments.

SKT was suspended in distilled water and intraduodenally administered to rats at doses of 0.5 or 1 g/kg through an indwelling catheter. LQ, LQG, LQA, ILQ, ILQG, ILQA, and GCM were dissolved in a mixture of ethanol, propylene glycol, and 0.9 % saline solution prepared in a ratio of 1:4:5. GA was dissolved in a mixture of ethanol, 25 % ammonia water, and 0.9 % saline solution prepared in a ratio of 1:0.05:8.95. Eight components [(LQ, LQG, LQA, ILQ, ILQG, ILQA, 20 μmol/kg), GCM (2.7 and 27 μmol/kg), and GA (7 and 35 μmol/kg)] were administered i.v. to rats. The rats received dantrolene sodium (Sigma-Aldrich, St. Louis, MO, USA) at doses of 10 and 30 mg/kg (i.d.).

### In vitro experiment on skeletal muscle

The left hemidiaphragm of male Wistar rats (300–400 g) was isolated and immediately mounted together with the phrenic nerve in a dissecting dish. Small muscle strips from the hemidiaphragm containing the innervating phrenic nerve were prepared and perfused with Krebs–Ringer bicarbonate buffer (113 mM NaCl, 5.0 mM KCl, 1.4 mM CaCl_2_, 0.9 mM MgSO_4_, 1.2 mM NaH_2_PO_4_, 25 mM NaHCO_3_, 11.5 mM glucose) oxygenated with 95 % O_2_ and 5 % CO_2_. Platinum electrodes were placed over the diaphragm muscles. A direct twitch response was elicited by stimulating the muscle supramaximally with 0.2-Hz rectangular pulses of 0.5-ms duration and recorded with an isometric transducer (Nihon Kohden).

SKT was suspended in Krebs–Ringer bicarbonate buffer at doses of 10^−5^–10^−3^ g/mL.

### Statistical analysis

All analytical data were processed using Analyst software, version 1.6.2 (AB SCIEX). The pharmacokinetics (PK) parameters, including peak plasma concentration (*C*_max_), time to *C*_max_ (*T*_max_), elimination half-life (*T*_1/2_), area under the curve (AUC_last_), and mean residence time (MRT), were calculated by non-compartmental analysis for sparse sampling using Phoenix WinNonlin software, version 6.3 (Certara, St. Louis, MO, USA). The calculated parameters are expressed as the mean or mean ± standard error of the mean (SE).

The results of experiments on skeletal muscle are expressed as mean ± SE. Changes in twitch height are expressed as a percentage of the original twitch height prior to the administration of each agent. Data were analyzed using one-way analysis of variance (ANOVA), followed by Student’s *t*-test or Dunnett’s test. *p*-Values of <0.05 were considered to be statistically significant.

## Results

### Pharmacokinetics study

**Fig. 1:**
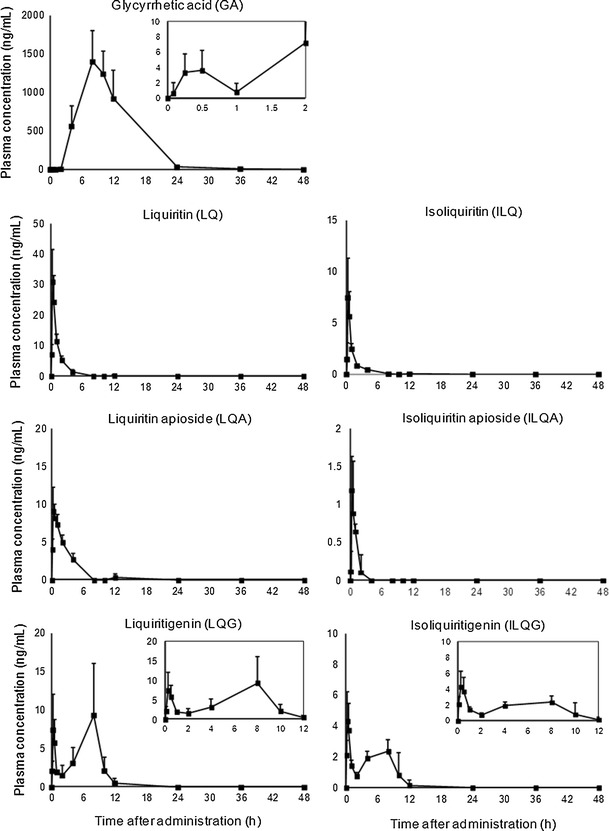
Plasma concentration profiles of 7 components of *G*. radix after a single oral administration of SKT at a dose of 1 g/kg. Data are expressed as mean + SD (*n* = 4–5 for each time point)

**Fig. 2:**
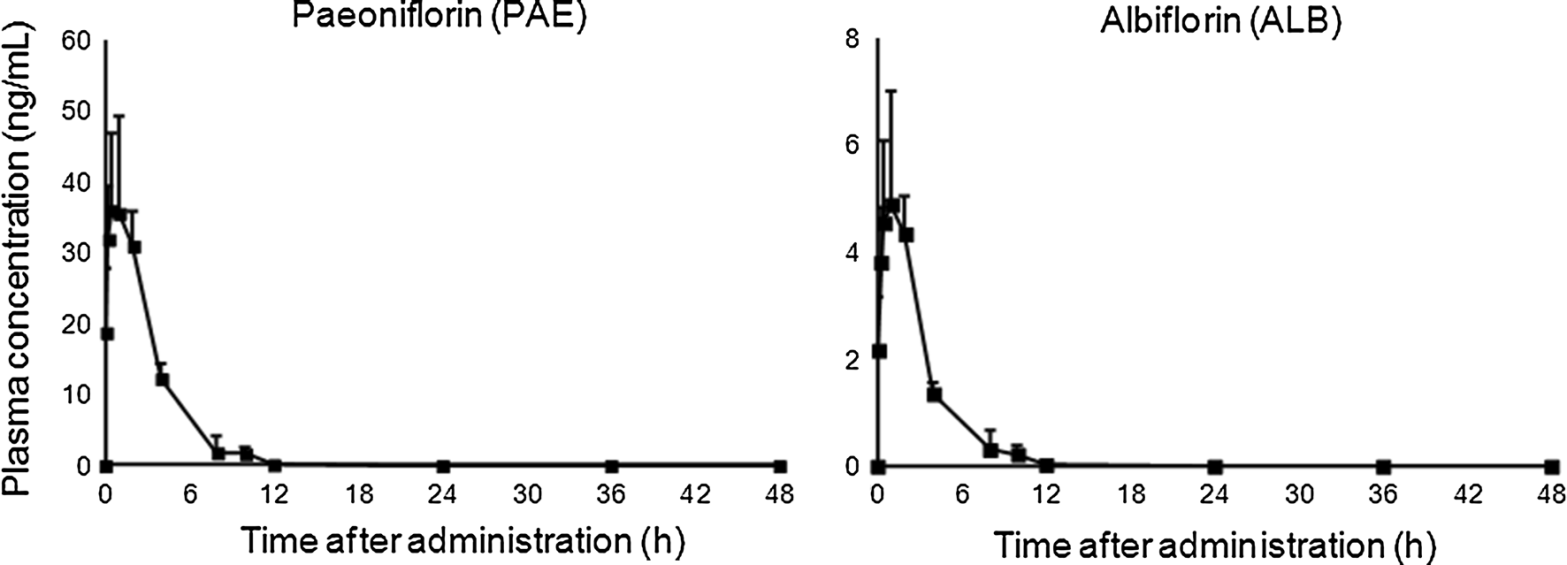
Plasma concentration profiles of 2 components of *P*. radix after a single oral administration of SKT at a dose of 1 g/kg. Data are expressed as mean + SD (*n* = 4–5 for each time point)

Plasma concentration–time profiles of the components after a single oral administration of SKT are presented in Figs. 1 and 2. The PK parameters are shown in Table 2.

**Table 2:** Pharmacokinetics parameters of SKT components after a single oral administration of SKT

Compound	*C*_max_ (ng/mL)	*T*_max_ (h)	AUC_last_ (h⋅ng/mL)	*T*_1/2_ (h)	MRT_last_ (h)
GA	1400 ± 181	8.00	15200 ± 1360	3.50	10.0
LQ	30.9 ± 4.70	0.250	37.2 ± 2.32	1.94	1.32
LQG	9.35 ± 3.01	8.00	50.0 ± 9.61	0.978	6.37
LQA	9.08 ± 1.43	0.250	27.1 ± 1.40	2.65	2.21
ILQ	7.47 ± 1.71	0.250	8.48 ± 0.621	1.61	1.50
ILQG	4.31 ± 0.866	0.250	19.3 ± 1.82	1.02	5.27
ILQA	1.18 ± 0.201	0.250	1.13 ± 0.138	0.469	0.723
PAE	36.0 ± 4.89	0.500	141 ± 7.43	1.48	2.50
ALB	4.88 ± 0.952	1.00	18.4 ± 1.09	1.55	2.51

The results are expressed as the mean or mean ± SE (*n* = 4–5 for each time point)

A previous study reported that orally administered glycyrrhizin (GL) was absorbed after being extensively metabolized to its aglycone, GA, by intestinal bacteria [13]. After SKT administration, GA was absorbed slowly, reached its maximum concentration at 8 h, and remained in the blood longer than other components.

LQ, ILQ, LQA, and ILQA had similar PK profiles, showing rapid absorption with a *T*_max_ of 0.25 h and an elimination half-life within 3 h. Plasma concentration–time course curves of two flavonoid aglycones (LQG and ILQG) exhibited bimodal kinetics with peaks at 0.25 and 8 h post dose. These phenomena were consistent with the findings of previous studies [14, 15]. Flavonoid glycosides (LQ, LQA, ILQ, and ILQA) have been found to be converted to their aglycones inside an organism [16]. LQG and ILQG remained in the blood for a prolonged period (10–12 h) due to delayed absorption of biotransformed LQG and ILQG. Plasma GCM was detected in only two rats at 0.25 and 4 h, respectively, at concentrations of less than 0.1 ng/mL (data not shown).

PAE and ALB were rapidly absorbed with peak plasma levels being reached within 1 h and eliminated rapidly with an approximate *T*_1/2_ of 1.5 h.

### Effects of SKT on skeletal muscle

**Fig. 3:**
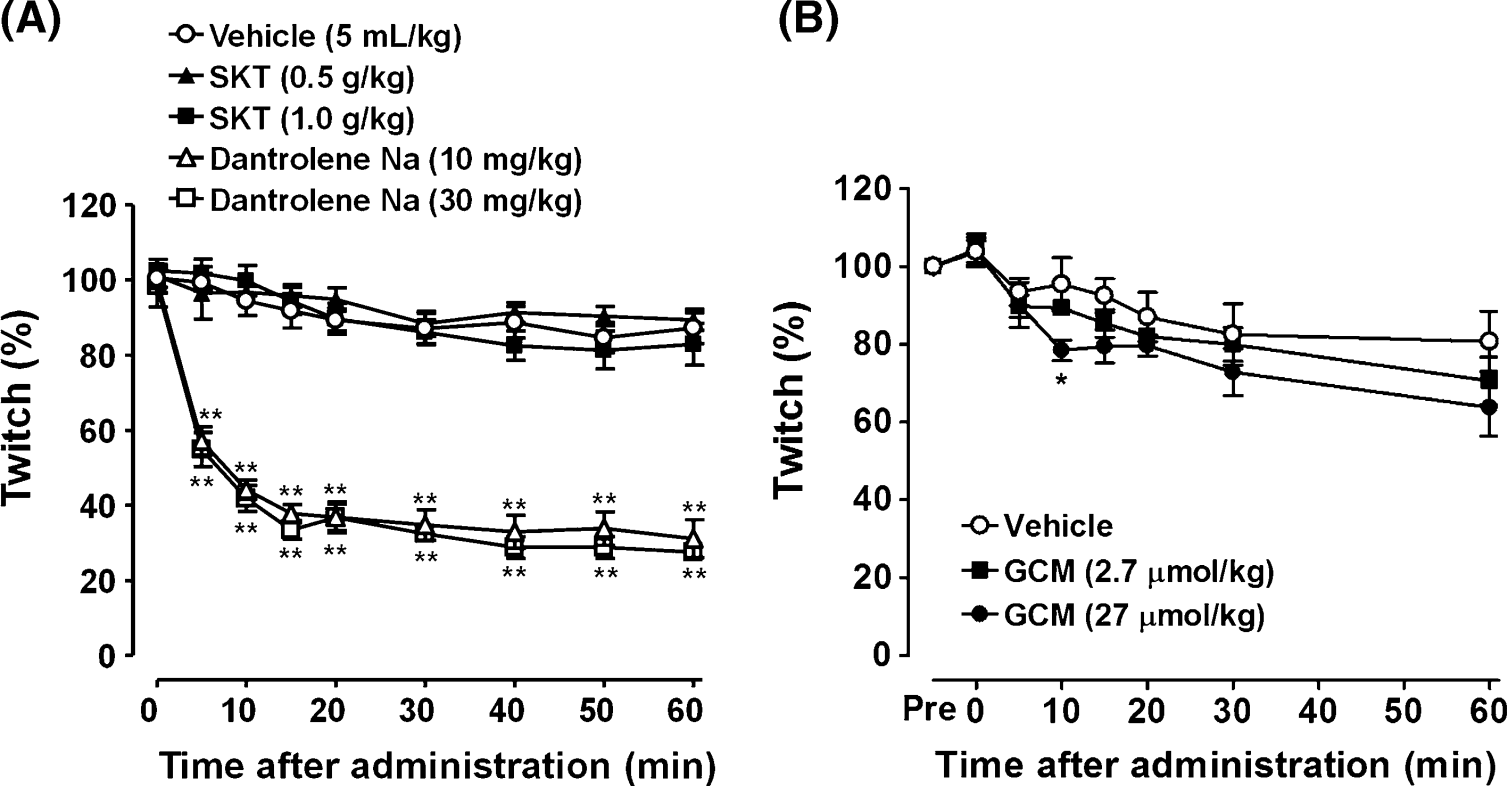
Effects of SKT (i.d.) and dantrolene sodium (i.d.) (**a**) and glycycoumarin (i.v.) (**b**) on electrically induced twitch contractions in a rat gastrocnemius muscle model. Each value represents the mean ± SE of 4–6 rats. *p < 0.05, **p < 0.01 compared with the vehicle group (Dunnett’s *t*-test)

The effects of SKT and dantrolene on rat gastrocnemius muscle are shown in Fig. 3a. SKT (0.5, 1 g/kg, i.d.) did not inhibit twitch contractions induced by electrical stimulation. In contrast, dantrolene (10, 30 mg/kg, i.d.) reduced twitch contractions by 43–72 % between 5 and 60 min after administration. There was no significant dose-dependent inhibitory effect of dantrolene. GCM at the highest dose tested (27 μmol/kg, i.v.) exhibited significant inhibition (17 % of the twitch amplitude) at 10 min after administration (Fig. 3b). The others, including flavonoids and triterpenoids, did not show inhibitory effects (Table 3). In in vitro experiments, SKT did not significantly inhibit the twitch response elicited by direct stimulation at doses of 10^−5^–10^−3^ g/mL (Table 4).

**Table 3:** Effects of flavonoids and triterpenoids on electrically induced twitch in rat gastrocnemius muscle

Treatment	Concentration	0 min	5 min	10 min	15 min	20 min	30 min	60 min
Vehicle	(μmol/kg)	102.1 ± 4.0	85.6 ± 4.5	80.2 ± 4.3	87.6 ± 11.1	82.6 ± 9.1	70.7 ± 6.4	68.5 ± 8.0
LQ	20	98.1 ± 3.2	91.6 ± 3.2	86.2 ± 3.7	83.0 ± 5.1	73.5 ± 8.5	70.4 ± 3.9	69.4 ± 3.4
ILQ	20	109.5 ± 27.2	92.5 ± 21.9	77.4 ± 6.8	77.8 ± 11.5	73.8 ± 13.1	67.7 ± 8.6	69.5 ± 10.8
LG	20	86.0 ± 3.4	77.1 ± 1.7	73.8 ± 2.6	67.6 ± 2.4	61.1 ± 2.8	56.9 ± 2.8	58.2 ± 5.5
ILG	20	106.0 ± 4.3	109.7 ± 10.0	100.1 ± 9.7	72.5 ± 8.1	70.7 ± 5.9	66.5 ± 9.3	69.6 ± 7.2
LQA	20	97.0 ± 5.7	86.2 ± 2.4	80.7 ± 4.1	72.0 ± 4.2	67.9 ± 4.2	66.5 ± 6.1	61.1 ± 3.7
ILQA	20	99.7 ± 6.4	87.9 ± 5.6	76.7 ± 2.0	69.8 ± 1.3	68.8 ± 2.8	64.6 ± 1.8	67.0 ± 3.5
GA	7	106.4 ± 6.4	94.5 ± 4.3	87.0 ± 4.6	80.1 ± 3.9	76.6 ± 5.3	74.0 ± 6.3	73.0 ± 8.0
GA	35	99.4 ± 7.1	84.6 ± 6.0	79.6 ± 5.2	78.8 ± 7.3	71.9 ± 6.3	67.4 ± 5.2	67.6 ± 6.3

Each value represents the mean ± SE of 3–6 rats. No statistically significant differences were found (Student’s *t*-test)

**Table 4:** Effects of SKT on electrically induced twitch in rat phrenic nerve-hemidiaphragm muscle preparation

	Concentration (g/mL)		
	1 × 10^−5^	1 × 10^−4^	1 × 10^−3^
Control	102.5 ± 5.3	111.3 ± 11.2	104.5 ± 9.6
SKT	104.5 ± 5.6	106.6 ± 8.3	96.1 ± 4.5

Each value represents the mean ± SE of 8 rats. No statistically significant differences were found (Student’s *t*-test)

## Discussion

Studies of the pharmacological mechanism of SKT in skeletal muscle have focused on two major components of *G*. radix and *P*. radix, GL and PAE, respectively. Basic pharmacological studies have shown that GL inhibits Ca^2+^-activated K^+^ channels, that PAE blocks intracellular Ca^2+^ movement, and that the combination of GL and PAE displays synergistic effects on antispasmodic activity at the myoneural junction [18–20]. In addition, six components of *G*. radix (GA, LQG, LQA, ILQG, ILQA, and GCM) were reported to significantly inhibit tetanic contractions after intravenous administration in rats [11]. Two components of *P*. radix (PAE and ALB) were found to have a significant antinociceptive effect via spinal α_2_-adrenoceptor activation [21, 22], although these components probably do not inhibit any contraction elicited by electrical stimulation because *P*. radix does not show a significant antispasmodic effect [11].

In our study, LQG, LQA, ILQG, ILQA, PAE, and ALB showed rapid absorption (*T*_max_ 0.25–1 h) after a single oral administration of 1 g/kg SKT to rats. GA was absorbed slowly, but it was detectable in plasma immediately after administration. Moreover, a clinical study previously reported that SKT alleviated painful muscle cramp within 15 min after oral administration [5]. Plasma concentration–time profiles of these components were clearly correlated with the rapid onset of the antispasmodic and analgesic effects of SKT on skeletal muscle in humans.

GA, LQG, and ILQG remained in the blood for a prolonged period. Symptoms of pseudoaldosteronism, such as increased blood pressure and edema, were observed in a double-blind study, although there were no significant differences in the frequency of adverse reactions between SKT and placebo groups. A study has also reported that 3-monoglucuronyl-glycyrrhetinic acid (3MGA) caused *G*. radix-induced pseudoaldosteronism [23]. Isbrucker and Burdock [24] reported that orally administered GL was hydrolyzed to 3MGA first and then to GA by intestinal bacteria. In the present study, GA showed very slow elimination from the systemic circulation, which suggests the possibility that repeated SKT administration increases the blood exposure level of GA. Therefore, it is necessary to exercise caution during multiple administration of SKT or combined application of other medicine containing *G*. radix or GL.

In the present study, we investigated the effects of SKT and its components on skeletal muscle contraction under physiological conditions in vivo and in vitro. SKT exhibited no inhibitory effects on electrically induced twitch contractions in a rat gastrocnemius muscle model in vivo, whereas dantrolene, a direct-acting peripheral muscle relaxant, robustly inhibited twitch contractions. A 1-g/kg dose of SKT is sufficient to inhibit tetanic contraction [11]. We also found that five *G*. radix isolates (GA, LQG, LQA, ILQG, and ILQA), which were responsible for the antispasmodic effect of SKT, and two flavanone glycoside forms (LQ and ILQ) exhibited no inhibitory effects on twitch contractions. Dantrolene concentrations (10 and 30 mg/kg) were set according to a previous study [25]. Lee et al. [11] reported that dantrolene did not inhibit muscle performance up to 90 mg/kg in the rotarod test in rats. In the present study, dantrolene robustly inhibited twitch contraction irrespective of concentration. This result is attributed to the fact that this experimental rat model exhibited greater sensitivity than the rotarod test reported in a previous study [11]. In addition, SKT did not show any inhibition, even at high concentrations, in an in vitro study. Notably, the majority of these results are contradictory to those of previous reports [11], i.e., SKT appeared to act specifically against tetanus rather than twitch, whereas dantrolene inhibited both tetanus and twitch contractions.

GCM (27 μmol/kg) at a higher concentration showed a transient inhibitory effect on the twitch contraction. Qiao et al. [15] reported that GCM was eliminated very quickly from the blood after oral administration of *G*. radix water extract (5 g/kg) in rats. Although GCM showed an immediate inhibitory effect on twitch contraction at 10 min after intravenous administration, we assumed that this effect disappeared with the decrease of the GCM level in the blood. When 1 g/kg of SKT was orally administered, we found that GCM was barely detectable in rat plasma. Therefore, we suggest that SKT did not significantly inhibit twitch contraction after oral administration. The observed plasma GCM levels after oral administration of SKT in humans are appealing, and a subsequent human PK study is currently in progress.

Ryanodine receptor (RyR)-mediated Ca^2+^ release from the sarcoplasmic reticulum (SR) is a crucial process in skeletal muscle contraction [26]. Previous studies reported that intracellular Ca^2+^ release mediated by RyR from the SR was involved in two different RyR1 opening modes, Ca^2+^-induced Ca^2+^ release (CICR) and physiological Ca^2+^ release [27–29]. Physiological Ca^2+^ release in skeletal muscle is caused by depolarization of the t-tubule membrane, whereas CICR is elicited by successive activation of calcium release from intracellular Ca^2+^ stores. In contractions induced by electrical stimulation, twitch and tetanus reflect physiological Ca^2+^ release and CICR, respectively, in their mechanisms of Ca^2+^ mobilization. In our study, SKT or its seven components did not inhibit twitch contractions, indicating that they selectively inhibited only CICR rather than physiological Ca^2+^ release. We suggest that this is the reason for SKT causing a lower incidence of muscle weakness in clinical use, although SKT is effective in reducing muscle spasms. RyR is recognized as the channel responsible for both CICR and physiological Ca^2+^ release, and it opens for each different mode, depending on the Ca^2+^ concentration around the SR or on the information to be transferred from the potential sensor of the t-tubule. Under physiological conditions, it is difficult to evoke CICR because Ca^2+^ cannot completely open the RyR channels. Therefore, to elucidate the mechanism of SKT, which selectively inhibited only CICR, it is necessary to measure the Ca^2+^ release from the SR using the system to induce CICR. In future, we are planning to study the inhibitory effect of SKT for caffeine, which induces CICR, or for clofibric acid, which induces Ca^2+^ release similar to physiological Ca^2+^ release [27], in skinned fibers.

Dantrolene depresses excitation–contraction coupling in skeletal muscle by inhibiting both CICR [30] and physiological Ca^2+^ release [31–34]; this is consistent with our results on dantrolene. At the molecular level, the dantrolene-binding site is located in the Leu^590^–Cys^609^ region of the N-terminal portion of the RyR1 domain switch [35]. Dantrolene or its analog has been found to stabilize a synthetic domain peptide (DP4) of RyR to block CICR from the SR or suppress the rate of RyR opening, respectively [35, 36]. It is an important issue for future study to determine whether SKT components bind to the same site as dantrolene; this may clarify the difference between dantrolene and SKT.

In summary, our findings indicated that SKT (up to 1 g/kg) and five *G*. radix isolates (GA, LQG, LQA, ILQG, and ILQA), which were responsible for the immediate antispasmodic efficacy of SKT, had no inhibitory effect on twitch tension caused by physiological Ca^2+^ release. This was in contrast to the effects of dantrolene, indicating that the mechanisms of muscle contraction of SKT and dantrolene in skeletal muscle differ. These findings suggest that SKT does not reduce the contractile force in skeletal muscle under physiological conditions, i.e., SKT may have a low risk of causing muscle weakness in clinical use. To prevent pseudoaldosteronism, it is necessary to exercise caution in the repeated administration of SKT or combined application of other medicine containing *G*. radix or GL. However, considering that most muscle relaxants and anticonvulsants cause various harmful side effects such as weakness and CNS depression, SKT appears to have a benign safety profile.
